# Crystal structure of penta­carbon­yl(2,2-di­fluoro­propane­thio­ato-κ*S*)manganese(I)

**DOI:** 10.1107/S2056989019004134

**Published:** 2019-04-02

**Authors:** Jean-Claude Daran, Roberto Morales-Cerrada, Christophe Fliedel, Florence Gayet, Vincent Ladmiral, Bruno Ameduri, Rinaldo Poli

**Affiliations:** a LCC-CNRS, Université de Toulouse, CNRS, INPT, Toulouse, France; b ICGM CNRS, Univ Montpellier, ENSCM, Montpellier, France

**Keywords:** crystal structure, Mn(CO)_5_ derivatives, Mn-S bond, inter­molecular contacts

## Abstract

The title compound, [Mn{SC(O)CF_2_CH_3_}(CO)_5_], has been isolated as a by-product during the reaction of K[Mn(CO)_5_] with CH_3_CF_2_COCl. It is built up from a di­fluoro­methyl­propane­thio­ate bonded to an Mn(CO)_5_ moiety through the S atom. The Mn atom has an almost perfect octa­hedral coordination sphere. It is one of the rare examples of compounds containing the (CO)_5_MnS—C fragment. In the crystal, the methyl group occupies a pocket surrounded by the O atoms of three carbonyl groups of the Mn(CO)_5_ moiety; however, the H⋯O distances are rather long. These inter­actions lead to the formation of layers lying parallel to (101), which enclose *R*_4_^4^(15) and *R*_4_^4^(16) ring motifs. The CF_2_ group is disordered over two sets of sites with occupancies of 0.849 (3) and 0.151 (3).

## Abstract 2

The synthesis and crystal structure of an unexpected CH_3_CF_2_C(O)SMn(CO)_5_ compound are described.

## Chemical context   

Alkyl­penta­carbonyl­manganese(I) complexes containing fluorinated alkyl groups, [Mn*R*_F_(CO)_5_], have been known since 1960 (Kaesz *et al.*, 1960 ▸; Beck *et al.*, 1961 ▸) but X-ray structures have been scarcely investigated until recently (Morales-Cerrada, Fliedel, Daran *et al.*, 2019 ▸). Our inter­est in these compounds is related to a study of the homolytic Mn—C bond strength and how this is affected by the F substitution at the α and β positions of the alkyl chain (Morales-Cerrada, Fliedel, Gayet *et al.*, 2019 ▸). The compounds where *R*_F_ stands for CH_2_CF_3_ and CF_2_CH_3_ may be considered as models for the role of [Mn(CO)_5_] as a radical-trapping species in the polymerization of vinyl­idene fluoride, where the Mn—C bonds may be formed and cleaved reversibly. While the synthesis of the CH_2_CF_3_ derivative could be accomplished as planned and the product could be obtained in a pure form and crystallized (Morales-Cerrada, Fliedel, Daran *et al.*, 2019 ▸), the synthesis of the CF_2_CH_3_ derivative led to the unexpected compound, [Mn{SC(O)CH_3_CF_2_}(CO)_5_] (1), reported here.
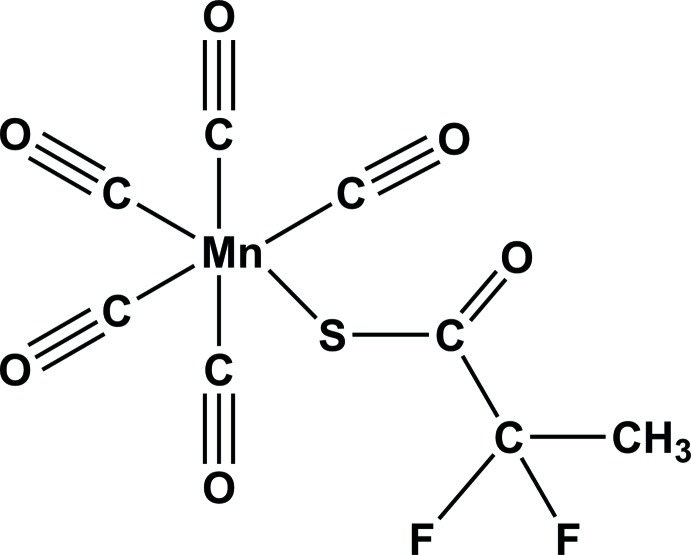


## Structural commentary   

**Figure 1:**
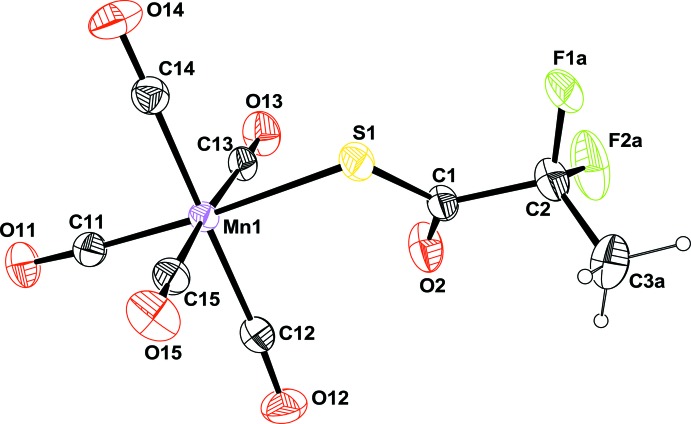
A view of the mol­ecular structure of compound (1), with the atom labelling. For clarity, only the major disordered component of the –CF_2_ group is shown. Displacement ellipsoids are drawn at the 50% probability level.

The title compound (1), is built up from a di­fluoro­methyl­propane­thio­ate bonded to an Mn(CO)_5_ moiety through the S atom (Fig. 1 ▸). Selected bond distances and bond angles involving atom Mn1 are given in Table 1 ▸, and it can be seen that this atom has a nearly perfect octa­hedral coordination sphere. As expected, the Mn1—S1—C1—C2 fragment is almost planar, as shown by the value of the torsion angle of −177.98 (11)°. This plane roughly bis­ects the dihedral angle formed by the C11/Mn1/C12/S1 and C11/Mn1/C13/S1 planes with values of 50.06 (7) and 39.9 (1)°, respectively, placing the O2 atom relatively close to the O atoms of the two carbonyl groups C12=O12 and C13=O13 with distances O2⋯O12 = 3.058 (2) Å and O2⋯O13 = 3.257 (2) Å. The smallest bond angles, 86.01 (5)° for C14—Mn1—S1 and 86.14 (5)° for C15—Mn1—S1, are certainly related to steric hindrance resulting from these relatively short intra­molecular O⋯O contacts. These short inter­actions might force the Mn1—S1 bond to bend slightly towards the equatorial plane [C12/C13/C14/C15]. The shortest Mn—C(O) distance is observed for the carbonyl group *trans* to the S atom; Mn1—C11 = 1.8376 (17) Å

## Supra­molecular features   

**Figure 2:**
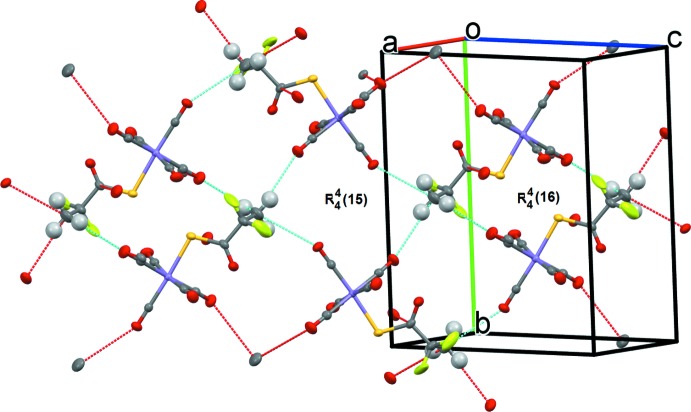
A view of the crystal packing of compound (1). The C—H⋯O inter­actions (Table 2 ▸) involving the major component of the disordered –CF_2_ group, are shown as dashed lines.

In the crystal, the methyl group occupies a pocket surrounded by O atoms of three carbonyl groups, C11=O11, C12=O12 and C14=O14, forming a two-dimensional network that develops parallel to (101); see Table 2 ▸ and Fig. 2 ▸. These rather weak C—H⋯O inter­actions result in the formation of two graph-set motifs, 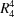
(15) and 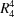
(16), as shown in Fig. 2 ▸.

## Database survey   

A search in the Cambridge Structural Database (CSD, V5.40, update February 2019; Groom *et al.*, 2016 ▸) using a (CO)_5_MnS—C fragment revealed only three hits. These include, [μ_2_-1,2-bis­(*p*-fluoro­phen­yl)-ethyl­ene-1,2-di­thiol­ato-*S,S′*]decacarbonyldi-manganese (CSD refcode CECCES; Lindner *et al.*, 1983 ▸), penta­carbonyl-[(*N*-penta­fluoro­thio)­fluoro­thio­form­imido-*S*]manganese (JEBNOT; Damerius *et al.*, 1989 ▸) and μ-1,2-di­thio­oxalatobis(penta­carbon­yl)manganese (TOXCMN; Weber & Mattes, 1979 ▸). The Mn—S, S—C, Mn—C bond distances and Mn—S—C bond angles are compared to those for compound (1) in Table 3 ▸. As in compound (1), the Mn—C bond *trans* to the S atom is significantly shorter than the four other Mn—C bonds. The longest Mn—S bond, 2.405 Å in CECCES, may be related to the presence of the bulky fluoro­phenyl group attached to the C(S) atom. For compound (1) and TOXCMN, both having an oxo group attached to the C(S) atom, the Mn—S—C angle is nearly identical, 106.26 (6) and *ca* 105.64°, respectively (Table 3 ▸). In contrast, this angle is slightly larger for CECCES and for JEBNOT, *ca* 108.8 and 108.1°, respectively.

## Synthesis and crystallization   

The synthesis of the target compound, [Mn(CF_2_CH_3_)(CO)_5_], requires transit through the corresponding acyl derivative, [Mn(COCF_2_CH_3_)(CO)_5_], because direct alkyl­ation of CH_3_CF_2_-*X* (*X* = Cl, Br) reagents by the powerful [Mn(CO)_5_]^−^ nucleophile suffers from the inverted polarity of the C—*X* bond, leading to [Mn*X*(CO)_5_] instead (Beck *et al.*, 1961 ▸). The corresponding acyl­ation using CH_3_CF_2_COCl as acyl­ating agent was successful (Morales-Cerrada, Fliedel, Daran *et al.*, 2019 ▸). However, the pure product could only be obtained when the 2,2-di­fluoro­propanoyl chloride was synthesized by the action of oxalyl chloride on 2,2-di­fluoro­propionic acid. In a first synthetic study, 2,2-di­fluoro­propionic acid was chlorin­ated by the more common thionyl chloride reagent, SOCl_2_. When the resulting acyl chloride was used to acyl­ate [Mn(CO)_5_]^−^, the title compound crystallized as colourless single crystals. The sulfur atom must have been provided by the thionyl chloride remaining as a contaminant in the acyl chloride reagent.

2,2-Di­fluoro­propanoyl chloride was freshly prepared as follows. To a 50 ml round flask equipped with a reflux condenser, was introduced 5.28 g of 2,2-di­fluoro­propionic acid (47.97 mmol) and 10.05 g of thionyl chloride (84.48 mmol; previously purified by reflux in the presence of sulfur powder and then distilled) was added dropwise. The mixture was then heated up to 363 K over 2 h (reflux). The product was purified by distillation (b.p. 308–313 K), giving 4.85 g of a colourless liquid. The amount of thionyl chloride contaminant in the distilled product could not be estimated by NMR spectroscopy.

**Synthesis of the title compound (1)**: To a Schlenk tube were introduced 390 mg (9.97 mmol) of metallic potassium and 358 mg (15.57 mmol) of metallic sodium under argon. They were crushed together to generate a liquid NaK alloy. A solution of dimanganese deca­carbonyl (2.00 g, 5.13 mmol) in 30 ml of dry THF was added and the resulting mixture was stirred for 3 h at room temperature, leading to the formation of K^+^[Mn(CO)_5_]^−^. The mixture was filtered through Celite to yield a greenish brown solution, rinsing the Celite with 10 ml of dry THF. Then, 2,2-tri­fluoro­propanoyl chloride (1.31 g, 10.19 mmol), made as described above, was added dropwise at room temperature. The resulting solution was further stirred at room temperature for 3 h, followed by evaporation of the solvents under reduced pressure. The product was purified by column chromatography through a silica gel column, using *n*-pentane as the mobile phase. After elimination of a first yellow fraction corresponding to [Mn_2_(CO)_10_], the mobile phase polarity was increased using a mixture of *n*-pentane and diethyl ether (2:1). An orange band was collected, followed by evaporation to dryness under reduced pressure to afford the product as an orange–brown liquid. The product was stored in the fridge (276–277 K), leading to the growth of thin colourless plate-like crystals of the title compound which were collected after two days.

## Refinement   

Crystal data, data collection and structure refinement details are summarized in Table 4 ▸. The methyl H atoms were fixed geometrically and treated as riding: C—H = 0.98 Å with *U*_iso_(H) = 1.5*U*_eq_(CH_3_). The two fluorine atoms presented elongated ellipsoids, which could be related to disorder. To consider a realistic chemical disorder, we defined a model by rotation around the C1—C2 bond. Initially, the model could be refined isotropically to define the occupancy factors using a free variable. The result showed a major component with an occupancy factor of 85% and a minor one at 15%. As a result, it was impossible to freely refine the thermal ellipsoids for the disordered CF_2_ group. The anisotropic refinement has been realized using severe EADP restraints for the C and F atoms.

## Supplementary Material

Crystal structure: contains datablock(s) 1, global. DOI: 10.1107/S2056989019004134/su5491sup1.cif

CCDC reference: 1906042

Additional supporting information:  crystallographic information; 3D view; checkCIF report

## Figures and Tables

**Table 1:** Selected geometric parameters (Å, °)

Mn1—C11	1.8376 (17)	Mn1—C14	1.8631 (17)
Mn1—C12	1.8807 (17)	Mn1—C15	1.8849 (17)
Mn1—C13	1.8720 (17)		
			
C11—Mn1—C12	91.08 (7)	C13—Mn1—C15	174.92 (7)
C11—Mn1—C13	90.69 (7)	C13—Mn1—S1	88.91 (5)
C11—Mn1—C14	90.47 (7)	C14—Mn1—C12	177.58 (7)
C11—Mn1—C15	94.32 (7)	C14—Mn1—C13	90.77 (7)
C11—Mn1—S1	176.45 (5)	C14—Mn1—C15	90.07 (7)
C12—Mn1—C15	87.96 (7)	C14—Mn1—S1	86.01 (5)
C12—Mn1—S1	92.46 (5)	C15—Mn1—S1	86.14 (5)
C13—Mn1—C12	91.07 (7)		

**Table 2:** Hydrogen-bond geometry (Å, °)

*D*—H⋯*A*	*D*—H	H⋯*A*	*D*⋯*A*	*D*—H⋯*A*
C3*A*—H3*A*1⋯O14^i^	0.98	2.81	3.732 (3)	158
C3*A*—H3*A*2⋯O12^ii^	0.98	2.79	3.753 (3)	166
C3*A*—H3*A*3⋯O11^iii^	0.98	2.79	3.564 (3)	136
C3*B*—H3*B*2⋯O12^ii^	0.98	2.81	3.777 (19)	168
C3*B*—H3*B*3⋯O11^iii^	0.98	2.37	3.165 (15)	138
C3*B*—H3*B*3⋯O11^iii^	0.98	2.37	3.165 (15)	138

Symmetry codes: (i) 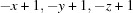; (ii) 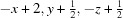; (iii) 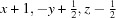.

**Table 3:** Comparison of selected bond lengths (Å) and bond angle (°) in the title compound (1) and related compounds having an Mn(CO)_5_SC fragment

Parameter	(1)	CECCES^*a*^	JEBNOT^*b*^	TOXCMN^*c*^
Mn—S	2.3768 (5)	2.405	2.384	2.379
C—S	1.725 (2)	1.741	1.723	1.737
Mn—S—C	106.26 (6)	108.84	108.12	105.64
Mn—C11	1.838 (2)	1.803	1.835	1.840
Mn—C12	1.881 (2)	1.867	1.871	1.883
Mn—C13	1.872 (2)	1.861	1.891	1.857
Mn—C14	1.863 (3)	1.864	1.871	1.880
Mn—C15	1.885 (2)	1.878	1.891	1.857

Notes: (*a*) Lindner *et al.* (1983 ▸); (*b*) Damerius *et al.* (1989 ▸); (*b*) Weber & Mattes (1979 ▸).

**Table 4:** Experimental details

Crystal data	
Chemical formula	[Mn(C_3_H_3_F_2_OS)(CO)_5_]
*M*_r_	320.10
Crystal system, space group	Monoclinic, *P*2_1_/*c*
Temperature (K)	173
*a*, *b*, *c* (Å)	6.3503 (4), 14.9583 (9), 12.3127 (9)
β (°)	97.149 (3)
*V* (Å^3^)	1160.49 (13)
*Z*	4
Radiation type	Mo *K*α
μ (mm^−1^)	1.36
Crystal size (mm)	0.40 × 0.26 × 0.04

Data collection	
Diffractometer	Nonius CAD-4 with APEXII CCD
Absorption correction	Multi-scan (Blessing, 1995 ▸)
*T*_min_, *T*_max_	0.621, 0.746
No. of measured, independent and observed [*I* > 2σ(*I*)] reflections	43723, 2554, 2261
*R*_int_	0.043
(sin θ/λ)_max_ (Å^−1^)	0.641

Refinement	
*R*[*F* ^2^ > 2σ(*F* ^2^)], *wR*(*F* ^2^), *S*	0.023, 0.062, 1.04
No. of reflections	2554
No. of parameters	175
No. of restraints	6
H-atom treatment	H-atom parameters constrained
Δρ_max_, Δρ_min_ (e Å^−3^)	0.55, −0.28

Computer programs: *APEX2* and *SAINT* (Bruker, 2014 ▸), *SIR97* (Altomare *et al.*, 1999 ▸), *SHELXL2014* (Sheldrick, 2015 ▸), *ORTEPIII* (Burnett & Johnson, 1996 ▸), *ORTEP-3 for Windows* (Farrugia, 2012 ▸) and *Mercury* (Macrae *et al.*, 2008 ▸).

## supplementary crystallographic information

### Crystal data

**Table:**

[Mn(C_3_H_3_F_2_OS)(CO)_5_]	*F*(000) = 632
*M**_r_* = 320.10	*D*_x_ = 1.832 Mg m^−^^3^
Monoclinic, *P*2_1_/*c*	Mo *K*α radiation, λ = 0.71073 Å
*a* = 6.3503 (4) Å	Cell parameters from 9996 reflections
*b* = 14.9583 (9) Å	θ = 2.2–28.9°
*c* = 12.3127 (9) Å	µ = 1.36 mm^−^^1^
β = 97.149 (3)°	*T* = 173 K
*V* = 1160.49 (13) Å^3^	Thin_plate, colourless
*Z* = 4	0.40 × 0.26 × 0.04 mm

### Data collection

**Table:**

Nonius CAD-4 with APEXII CCD diffractometer	2554 independent reflections
Radiation source: fine-focus sealed tube	2261 reflections with *I* > 2σ(*I*)
Graphite monochromator	*R*_int_ = 0.043
φ and ω scans	θ_max_ = 27.1°, θ_min_ = 2.7°
Absorption correction: multi-scan (Blessing, 1995)	*h* = −8→8
*T*_min_ = 0.621, *T*_max_ = 0.746	*k* = −19→19
43723 measured reflections	*l* = −15→15

### Refinement

**Table:**

Refinement on *F*^2^	Primary atom site location: structure-invariant direct methods
Least-squares matrix: full	Secondary atom site location: difference Fourier map
*R*[*F*^2^ > 2σ(*F*^2^)] = 0.023	Hydrogen site location: inferred from neighbouring sites
*wR*(*F*^2^) = 0.062	H-atom parameters constrained
*S* = 1.04	*w* = 1/[σ^2^(*F*_o_^2^) + (0.033*P*)^2^ + 0.5266*P*] where *P* = (*F*_o_^2^ + 2*F*_c_^2^)/3
2554 reflections	(Δ/σ)_max_ = 0.001
175 parameters	Δρ_max_ = 0.55 e Å^−^^3^
6 restraints	Δρ_min_ = −0.28 e Å^−^^3^

### Special details

**Table:**

Geometry. All esds (except the esd in the dihedral angle between two l.s. planes) are estimated using the full covariance matrix. The cell esds are taken into account individually in the estimation of esds in distances, angles and torsion angles; correlations between esds in cell parameters are only used when they are defined by crystal symmetry. An approximate (isotropic) treatment of cell esds is used for estimating esds involving l.s. planes.

### Fractional atomic coordinates and isotropic or equivalent isotropic displacement parameters (Å^2^)

**Table:**

	*x*	*y*	*z*	*U*_iso_*/*U*_eq_	Occ. (<1)
Mn1	0.43292 (4)	0.29392 (2)	0.45193 (2)	0.01827 (8)	
S1	0.53795 (7)	0.42961 (3)	0.37381 (4)	0.02705 (11)	
O2	0.6662 (2)	0.32546 (9)	0.22242 (11)	0.0380 (3)	
O11	0.2678 (2)	0.13030 (8)	0.54815 (10)	0.0335 (3)	
O12	0.8262 (2)	0.19606 (9)	0.40668 (12)	0.0386 (3)	
O13	0.1947 (2)	0.24358 (10)	0.23502 (10)	0.0374 (3)	
O14	0.0554 (2)	0.39501 (10)	0.50870 (13)	0.0429 (3)	
O15	0.6894 (2)	0.35866 (9)	0.65865 (11)	0.0361 (3)	
C1	0.6470 (3)	0.40015 (11)	0.25739 (14)	0.0258 (3)	
C11	0.3361 (3)	0.19238 (11)	0.51356 (13)	0.0231 (3)	
C12	0.6773 (3)	0.23362 (11)	0.41940 (14)	0.0249 (3)	
C13	0.2873 (3)	0.26287 (11)	0.31555 (14)	0.0246 (3)	
C14	0.1982 (3)	0.35751 (11)	0.48685 (14)	0.0264 (3)	
C15	0.5915 (3)	0.33322 (10)	0.58282 (14)	0.0238 (3)	
C2	0.7351 (3)	0.47775 (12)	0.19361 (16)	0.0348 (4)	
C3A	0.9645 (4)	0.4925 (2)	0.2222 (2)	0.0413 (6)	0.849 (3)
H3A1	0.993454	0.510299	0.299223	0.062*	0.849 (3)
H3A2	1.011515	0.539893	0.175822	0.062*	0.849 (3)
H3A3	1.041157	0.437149	0.210635	0.062*	0.849 (3)
F1A	0.6207 (3)	0.55205 (11)	0.2005 (2)	0.0672 (7)	0.849 (3)
F2A	0.6999 (3)	0.45454 (13)	0.08233 (12)	0.0604 (6)	0.849 (3)
C3B	0.952 (2)	0.4766 (13)	0.1799 (15)	0.0413 (6)	0.151 (3)
H3B1	1.037235	0.468084	0.251102	0.062*	0.151 (3)
H3B2	0.990454	0.533469	0.148047	0.062*	0.151 (3)
H3B3	0.979264	0.427449	0.130863	0.062*	0.151 (3)
F1B	0.7158 (19)	0.5589 (6)	0.2621 (11)	0.0672 (7)	0.151 (3)
F2B	0.5979 (18)	0.4975 (8)	0.1138 (8)	0.0604 (6)	0.151 (3)

### Atomic displacement parameters (Å^2^)

**Table:**

	*U*^11^	*U*^22^	*U*^33^	*U*^12^	*U*^13^	*U*^23^
Mn1	0.01945 (13)	0.01603 (13)	0.01948 (13)	0.00083 (8)	0.00298 (9)	0.00078 (9)
S1	0.0315 (2)	0.01629 (19)	0.0345 (2)	0.00001 (15)	0.00874 (18)	0.00293 (16)
O2	0.0568 (9)	0.0261 (7)	0.0341 (7)	−0.0052 (6)	0.0176 (6)	0.0000 (5)
O11	0.0430 (7)	0.0257 (6)	0.0326 (7)	−0.0076 (5)	0.0081 (6)	0.0041 (5)
O12	0.0318 (7)	0.0426 (8)	0.0422 (8)	0.0146 (6)	0.0076 (6)	0.0002 (6)
O13	0.0402 (7)	0.0454 (8)	0.0250 (7)	−0.0088 (6)	−0.0029 (6)	0.0005 (6)
O14	0.0294 (7)	0.0434 (8)	0.0570 (9)	0.0104 (6)	0.0106 (6)	−0.0062 (7)
O15	0.0456 (8)	0.0279 (7)	0.0319 (7)	−0.0035 (6)	−0.0072 (6)	−0.0033 (5)
C1	0.0239 (8)	0.0256 (8)	0.0276 (8)	−0.0028 (6)	0.0021 (6)	0.0077 (7)
C11	0.0254 (8)	0.0241 (8)	0.0197 (8)	0.0009 (6)	0.0028 (6)	−0.0022 (6)
C12	0.0285 (9)	0.0223 (8)	0.0238 (8)	−0.0002 (7)	0.0025 (6)	0.0010 (6)
C13	0.0263 (8)	0.0217 (8)	0.0266 (9)	−0.0010 (6)	0.0069 (7)	0.0047 (6)
C14	0.0265 (8)	0.0247 (8)	0.0279 (9)	−0.0010 (7)	0.0026 (7)	0.0008 (7)
C15	0.0270 (8)	0.0163 (8)	0.0286 (8)	0.0014 (6)	0.0053 (7)	0.0018 (6)
C2	0.0370 (10)	0.0293 (9)	0.0384 (10)	−0.0031 (8)	0.0065 (8)	0.0118 (8)
C3A	0.0401 (12)	0.0418 (16)	0.0438 (18)	−0.0138 (10)	0.0117 (13)	0.0001 (13)
F1A	0.0639 (12)	0.0365 (8)	0.1106 (19)	0.0244 (9)	0.0482 (12)	0.0455 (11)
F2A	0.0864 (14)	0.0664 (12)	0.0267 (8)	−0.0336 (10)	0.0003 (8)	0.0110 (7)
C3B	0.0401 (12)	0.0418 (16)	0.0438 (18)	−0.0138 (10)	0.0117 (13)	0.0001 (13)
F1B	0.0639 (12)	0.0365 (8)	0.1106 (19)	0.0244 (9)	0.0482 (12)	0.0455 (11)
F2B	0.0864 (14)	0.0664 (12)	0.0267 (8)	−0.0336 (10)	0.0003 (8)	0.0110 (7)

### Geometric parameters (Å, º)

**Table:**

Mn1—C11	1.8376 (17)	C1—C2	1.545 (2)
Mn1—C12	1.8807 (17)	C2—F2B	1.264 (9)
Mn1—C13	1.8720 (17)	C2—F1A	1.336 (2)
Mn1—C14	1.8631 (17)	C2—F2A	1.404 (3)
Mn1—C15	1.8849 (17)	C2—C3B	1.409 (13)
Mn1—S1	2.3768 (5)	C2—C3A	1.472 (3)
S1—C1	1.7250 (18)	C2—F1B	1.492 (11)
O2—C1	1.209 (2)	C3A—H3A1	0.9800
O11—C11	1.131 (2)	C3A—H3A2	0.9800
O12—C12	1.127 (2)	C3A—H3A3	0.9800
O13—C13	1.126 (2)	C3B—H3B1	0.9800
O14—C14	1.127 (2)	C3B—H3B2	0.9800
O15—C15	1.122 (2)	C3B—H3B3	0.9800
			
C11—Mn1—C12	91.08 (7)	F1A—C2—F2A	104.25 (18)
C11—Mn1—C13	90.69 (7)	F2B—C2—C3B	119.9 (9)
C11—Mn1—C14	90.47 (7)	F1A—C2—C3A	112.9 (2)
C11—Mn1—C15	94.32 (7)	F2A—C2—C3A	107.65 (19)
C11—Mn1—S1	176.45 (5)	F2B—C2—F1B	98.7 (7)
C12—Mn1—C15	87.96 (7)	C3B—C2—F1B	103.2 (8)
C12—Mn1—S1	92.46 (5)	F2B—C2—C1	108.2 (4)
C13—Mn1—C12	91.07 (7)	F1A—C2—C1	110.99 (16)
C13—Mn1—C15	174.92 (7)	F2A—C2—C1	106.65 (15)
C13—Mn1—S1	88.91 (5)	C3B—C2—C1	118.3 (8)
C14—Mn1—C12	177.58 (7)	C3A—C2—C1	113.66 (18)
C14—Mn1—C13	90.77 (7)	F1B—C2—C1	105.3 (4)
C14—Mn1—C15	90.07 (7)	C2—C3A—H3A1	109.5
C14—Mn1—S1	86.01 (5)	C2—C3A—H3A2	109.5
C15—Mn1—S1	86.14 (5)	H3A1—C3A—H3A2	109.5
C1—S1—Mn1	106.26 (6)	C2—C3A—H3A3	109.5
O2—C1—C2	117.03 (16)	H3A1—C3A—H3A3	109.5
O2—C1—S1	126.92 (13)	H3A2—C3A—H3A3	109.5
C2—C1—S1	116.02 (13)	C2—C3B—H3B1	109.5
O11—C11—Mn1	176.70 (15)	C2—C3B—H3B2	109.5
O12—C12—Mn1	175.65 (15)	H3B1—C3B—H3B2	109.5
O13—C13—Mn1	177.98 (15)	C2—C3B—H3B3	109.5
O14—C14—Mn1	179.09 (17)	H3B1—C3B—H3B3	109.5
O15—C15—Mn1	177.59 (15)	H3B2—C3B—H3B3	109.5
			
Mn1—S1—C1—C2	−177.98 (11)		

### Hydrogen-bond geometry (Å, º)

**Table:**

*D*—H···*A*	*D*—H	H···*A*	*D*···*A*	*D*—H···*A*
C3*A*—H3*A*1···O14^i^	0.98	2.81	3.732 (3)	158
C3*A*—H3*A*2···O12^ii^	0.98	2.79	3.753 (3)	166
C3*A*—H3*A*3···O11^iii^	0.98	2.79	3.564 (3)	136
C3*B*—H3*B*2···O12^ii^	0.98	2.81	3.777 (19)	168
C3*B*—H3*B*3···O11^iii^	0.98	2.37	3.165 (15)	138
C3*B*—H3*B*3···O11^iii^	0.98	2.37	3.165 (15)	138

Symmetry codes: (i) −*x*+1, −*y*+1, −*z*+1; (ii) −*x*+2, *y*+1/2, −*z*+1/2; (iii) *x*+1, −*y*+1/2, *z*−1/2.
